# Associations between oral health problems and inflammatory bowel disease: evidence from prospective cohort study

**DOI:** 10.3389/fimmu.2026.1751956

**Published:** 2026-03-12

**Authors:** Yixiao Wang, Xiuxiu Sun, Jiahao Chen, Tong Wang, Wanchun Wang, Dongfeng Zhang

**Affiliations:** 1 Qingdao Stomatological Hospital Affiliated to Qingdao University, Qingdao, China; 2 School of Public Health, Qingdao University, Qingdao, China

**Keywords:** comorbidity, dental disease, epidemiology, inflammatory bowel disease, inflammatory responses, oral health

## Abstract

**Background:**

An association exists between oral health problems and inflammatory bowel disease (IBD). However, few studies have considered comprehensive oral health problems and the concurrent presence of multiple oral health problems in relation to IBD. Additionally, the role of inflammatory responses as a mediator in this relationship remains unexplored. This study aimed to investigate association between oral health problems and IBD utilizing data from the UK Biobank.

**Methods:**

Oral health were assessed via self-reported. IBD was defined by disease classification codes from the International Classification of Diseases-10. Cox regression models were employed to analyze the relationships between oral health problems and IBD, using mediation analysis to investigate the role and effect size of inflammatory indicators in this association.

**Results:**

Among 412,134 participants, mouth ulcers (HR: 1.25, 95% CI: 1.02-1.53), painful gums (HR: 1.50, 95% CI: 1.12-2.03), and dentures (HR: 1.30, 95% CI: 1.10-1.53) linked to Crohn’s disease (CD) risk. Mouth ulcers (HR: 1.16, 95% CI: 1.01-1.34) and dentures (HR: 1.18, 95% CI: 1.05-1.33) linked to ulcerative Colitis (UC) risk. Having two (HR: 1.29, 95% CI: 1.02-1.63)/three (HR: 1.69, 95% CI: 1.14-2.49)/more than three (HR: 2.13, 95% CI: 1.20-3.78) oral health problems correlated with CD risk, with more oral health problems correlating with higher CD risk (P for trend < 0.001). Low-grade inflammation index (INFLA-score) positively mediated the association between painful gums (proportion mediated=3.09%)/dentures (proportion mediated=5.40%) and CD.

**Conclusions:**

Mouth ulcers and dentures may increase the risk of CD and UC, while painful gums associates with CD. More oral health problems link to higher CD risk. Inflammatory responses partially mediating the association between painful gums/dentures and CD.

## 1 Introduction

Inflammatory bowel disease (IBD), comprising ulcerative colitis (UC) and Crohn’s disease (CD), features chronic and relapsing gastrointestinal inflammation (1). The etiology of IBD is multifactorial, including lifestyle, environmental factors, immune-inflammatory response and microbial dysbiosis (2, 3). The chronic progression of IBD not only results to a notable deterioration in quality of patients’ life but also places a substantial burden on global health (4, 5). Therefore, early intervention to address modifiable lifestyle and environmental risk factors is critical for reducing the incidence of IBD.

Oral health serves as a crucial indicator of systemic health (6). Oral health problems comprise a spectrum of chronic conditions affecting oral tissues, primarily including mouth ulcers, painful gums, bleeding gums, tooth loss, toothache and dentures (7, 8). Oral health problems typically present as comorbidities rather than in isolation, with these problems influencing gastrointestinal health. In recent years, accumulating evidence has revealed a substantial association between oral health and gastrointestinal diseases (9, 10). Studies have suggested that oral health problems may trigger to the migration of derived pathogens, impairing intestinal function and disturbing immune balance (11, 12), meanwhile also inducing systemic inflammatory responses (13, 14). All of these factors are key to the pathogenesis of IBD (15, 16). Thus, inflammatory responses may serve as a mediator connecting the latter two.

IBD has been linked to oral health problems in previous research. Most studies on oral health and IBD are cross-sectional. Previous studies have identified that oral health problems may elevate IBD risk in specific populations, including the homeless with severe mental illness (Mejia-Lancheros et al.) and women with poor self-reported oral health (Kato et al.) (17–19). Notably, a cohort study in Sweden (N = 20,162) revealed protective effects of tooth loss, dental plaque and oral mucosal lesions against the development of IBD (20). Additionally, a Finnish cohort study reported no significant association between oral health and IBD (21). Current research mainly focus on the impact of periodontitis on IBD, including one longitudinal study based on the U.S. population (22),and several studies based on Asian populations (23–25).

However, there are still unresolved questions concerning the association between oral health and IBD. Previous research has been restricted to few oral health problem and lacked a systematic assessment of comprehensive problems in relation to IBD. Meanwhile, evidence remains conflicting, particularly between cohort and cross-sectional studies, as well as among cohort studies themselves. Furthermore, previous studies have only considered the relationship between individual oral health problems and IBD, without examining the impact of number of oral health problems on IBD. Finally, prospective studies investigating the mediating role and effect size of specific inflammatory factors in the association between oral health problems and IBD have not been definitively examined.

Therefore, we aimed to explore the longitudinal associations of multiple self-reported oral health problems (including oral ulcers, painful gums, bleeding gums, loose teeth, toothache and dentures)/number of oral health problems with IBD, utilizing prospective cohort data. Furthermore, we also investigated mediating role and effect size of inflammatory indicators in connections relating oral health problems to IBD.

## 2 Materials and methods

### 2.1 Study population and design

The UK Biobank study had received ethical approval from the Northwest Multi-Centre Research Ethics Committee (11/NW/0382). All participants have submitted written informed consent.

Data from the UK Biobank, a large-scale biomedical database with 502,370 participants (aged 37–73 years) recruited during 2006-2010, was utilized in this prospective cohort study. Baseline assessments included comprehensive lifestyle and health evaluations, along with biospecimen collection (whole blood, saliva and urine) (26). Health outcomes were obtained through national healthcare registry linkages (26).

**Figure 1:**
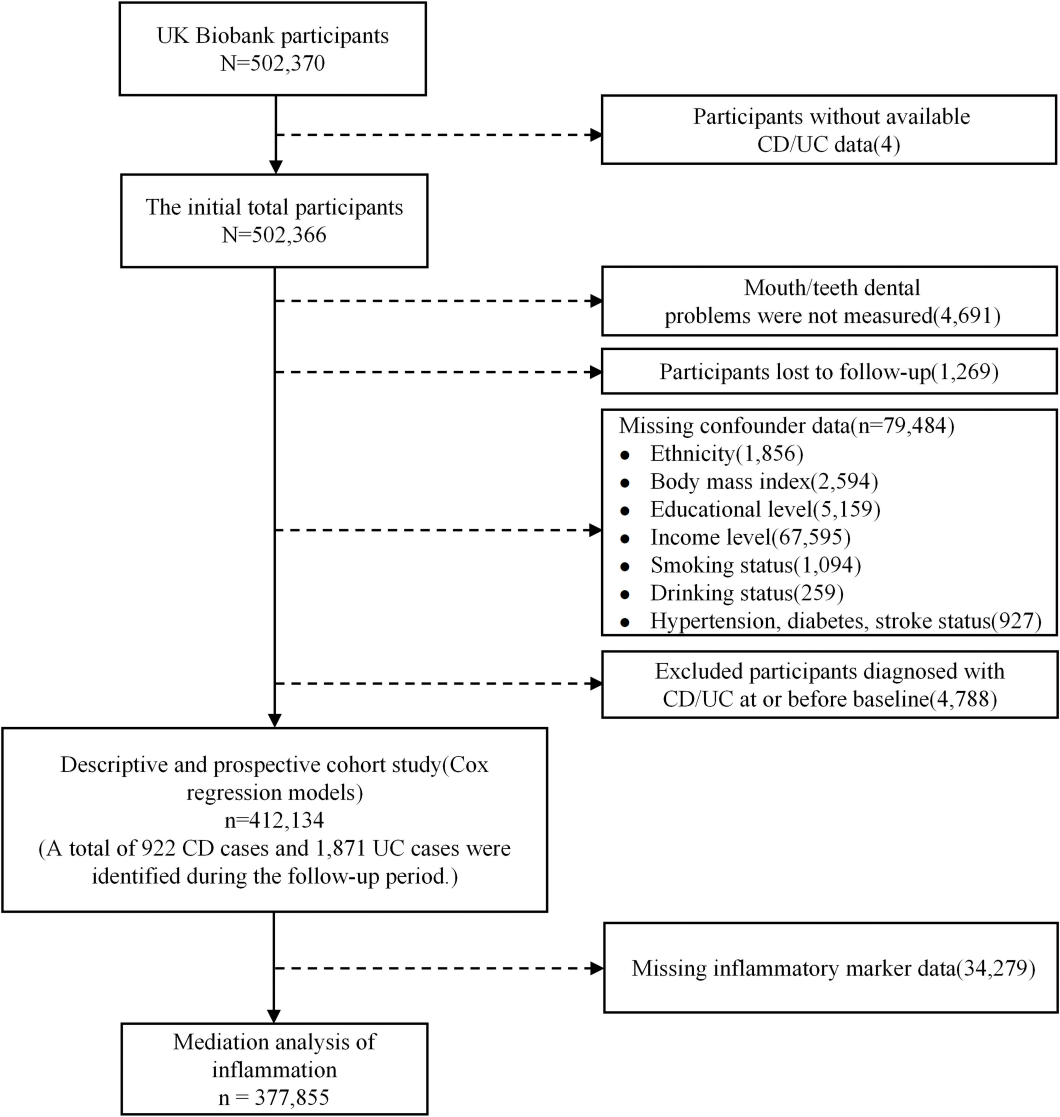
Flow diagram of study participant selection. CD, Crohn’s disease; UC, ulcerative Colitis.

From the overall UK Biobank population of 502,370 participants, we excluded those without available CD/UC data, resulting in an initial inclusion of 502,366 participants. Prior to conducting descriptive statistics and the cohort analysis, we excluded participants with the response “Prefer not to answer” to any oral health question, those with incomplete covariate data, and those diagnosed with CD/UC at or before the start of follow-up. After these exclusions, 412,134 participants remained for the main analysis. Furthermore, for the mediation analysis, participants with missing data on inflammatory markers were excluded from this established cohort. The detailed inclusion and exclusion criteria are presented in **Figure 1**.

### 2.2 Exposures

#### 2.2.1 Oral health problems

This study enrolled six self-reported oral health problems among participants from UK Biobank, which were obtained via a touchscreen questionnaire at baseline. The specific conditions included mouth ulcers, painful gums, bleeding gums, loose teeth, toothache and dentures, which were collected by self-reported data: “Do you have any of the following conditions? (You can select multiple answers)”. Before analyzing, participants who declined to answer this question were excluded from the study.

#### 2.2.2 Number of oral health problems

In the study, we counted the number of oral health problems reported by participants. Given the relatively few data in the higher count categories, participants reporting more than three oral health problems were combined into a single group to maintain adequate statistical power and ensure stable effect estimates. Participants were categorized by the count of oral health problems into four groups (one, two, three and more than three health problems). For the analysis, participants without any oral health problems were included in the reference group to examine the impact of the number of oral health problem on IBD. Furthermore, tests for trend were also incorporated in the study, modeling the number of oral health problems as a one degree-of-freedom linear term.

### 2.3 Outcomes

This study focused on new-onset IBD, including both CD and UC, as its main endpoint. The identification of IBD cases utilized diagnostic codes from the International Classification of Diseases Tenth editions (ICD-10) (K50 for CD and K51 for UC) and ICD-9 (555 for CD and 556 for UC), combined with dedicated codes for primary care and self-reported data. The identification of IBD cases involved synthesizing data from multiple sources: primary care data, death register records, hospital inpatient information and self-reported data, all converted to ICD-10 codes. Participants were considered to have IBD if any single data source showed positive identification. The follow-up period concluded at the earliest occurrence among the hospital record date, loss to follow-up, death, or the study cutoff date (31 December 2022) of IBD. In analyses, we used the first recorded IBD diagnosis during follow-up as the outcome date.

### 2.4 Mediators

The inflammatory marker analyzed in this study were sourced from baseline blood tests in the UK Biobank cohort. The low-grade inflammation index (INFLA-score), a combined metric including C-reactive protein (CRP) levels, white blood cell count (WBC), platelet count and neutrophil-to-lymphocyte ratio (NLR=Neutrophil/Lymphocyte), encompasses various elements of the inflammatory responses process (27). In contrast to individual indicators, the INFLA-score combines multiple inflammatory factors, resulting in a more reliable measure of systemic inflammation (28). In the present study, we selected the INFLA-score to evaluate the mediating role of inflammatory responses in the relationship between poor oral health and IBD. The INFLA-score assigned each biomarker from -4 to +4 based on its decile distributions. Specifically, values in upper deciles (7th to 10th) were assigned +1 to +4 points; in middle deciles (5th and 6th) counted as 0 points; and those in lower deciles (1st to 4th) were awarded -4 to -1 points. The INFLA-score ranged from -16 to 16, which higher values corresponded to elevated low-grade inflammation (29, 30).

### 2.5 Covariates

Baseline covariates were collected through the touchscreen questionnaire, including sociodemographic (age, sex, ethnicity, education level, income level), body size measures (body mass index [BMI]), lifestyle characteristics (smoking status, drinking status) and health and medical history (hypertension, diabetes, stroke status) (20, 24).

### 2.6 Statistical analyses

Descriptive statistics characterized participants’ baseline profiles and self-reported oral health problems. Categorical variables were presented as absolute frequencies with percentages.

In this cohort study, all participants without IBD at baseline were followed from enrollment until IBD diagnosis, loss to follow-up, death, or the study cutoff date (31 December 2022), whichever of these took place first. We used Cox proportional hazards regression to estimate associations between oral health problems/count of comorbidities and CD/UC, and results were expressed as hazard ratios (HRs) with 95% confidence intervals (CIs) using person-years as the timescale. The minimally adjusted model controlled for baseline age, sex, ethnicity, education level, income level, BMI. The fully adjusted model additionally incorporated smoking status, drinking status, hypertension, diabetes and stroke. Schoenfeld residual tests confirmed proportionality assumptions.

We performed stratified analyses by median age (<58 and ≥58 years), sex, smoking status and drinking status. The study’s robustness was examined through three sensitivity analyses. First, to mitigate reverse causation, we excluded participants who were diagnosed with IBD with ≤2 years of follow-up. Second, given the established association between colorectal cancer and IBD, we excluded participants diagnosed with colorectal cancer during follow-up. Third, we performed multiple imputation for covariates with missing values using the mice package in R.

To investigate the mediating role of INFLA in the association between oral health problems and IBD, we conducted mediation analysis in R using the “mediation package” with bootstrap (1,000 iterations) (31). Based on fully adjusted models, we estimated the direct effect (oral health problems’ independent effect on CD/UC), indirect effect (mediation via inflammatory factors), total effect and mediation proportion (the percentage of total effect accounted for by mediation). Results were presented as percentages or indirect effect estimates, accompanied by 95% CIs.

All data processing and statistical analyses were performed using R (version 4. 3. 3), with statistical significance defined as p<0. 05 (two-tailed).

## 3 Results

### 3.1 Baseline characteristics

Among 412,134 participants from UK Biobank (mean age 56.15 ± 8.08 years, 52.7% female), 41,770 (10.1%) were of mouth ulcers, 12204 (3.0%) of painful gums, 55959 (13.6%) of bleeding gums, 17481 (4.2%) of loose teeth, 18325 (4.4%) of toothaches and 64722 (15.7%) of dentures. Baseline characteristics compared between participants with and without oral health problems are presented in **Table 1**. Participants with oral health problems showed a greater tendency to be older, female, smokers, obesity and a higher incident of comorbidities (P<0.0001 for each variable).

**Table 1:** Baseline characteristics of participants according to presence of oral health problems.

Characteristic	All (N = 412,134)	Oral health problems (-) (N = 251,574)	Oral health problems (+) (N =160,560)	p-Value
Age at baseline, years				<0.001***
<45	44,847 (10.9%)	29,773 (11.8%)	15,074 (9.4%)	
45-54	122,301 (29.7%)	79,521 (31.6%)	42,780 (26.6%)	
55-64	172,517 (41.9%)	104,034 (41.4%)	68,483 (42.7%)	
≥65	72,469 (17.6%)	38,246 (15.2%)	34,223 (21.3%)	
Sex				<0.001***
Female	217,089 (52.7%)	130,442 (51.9%)	86,647 (54.0%)	
Male	195,045 (47.3%)	121,132 (48.1%)	73,913 (46.0%)	
Ethnicity				<0.001***
White	393,010 (95.4%)	241,021 (95.8%)	151,989 (94.7%)	
Mixed	5,498 (1.3%)	3,208 (1.3%)	2,290 (1.4%)	
Asian or Asian British	7,792 (1.9%)	4,221 (1.7%)	3,571 (2.2%)	
Black or Black British	5,834 (1.4%)	3,124 (1.2%)	2,710 (1.7%)	
Education				<0.001***
College or University degree	144,794 (35.1%)	96,655 (38.4%)	48,139 (30.0%)	
Others	267,340 (64.9%)	154,919 (61.6%)	112,421 (70.0%)	
Income levels				<0.001***
Level 1 (<£18,000)	92,527 (22.5%)	46,182 (18.4%)	46,345 (28.9%)	
Level 2 (£8,000–30,999)	104,833 (25.4%)	61,686 (24.5%)	43,147 (26.9%)	
Level 3 (£31,000–52,000)	107,996 (26.2%)	69,577 (27.7%)	38,419 (23.9%)	
Level 4 (>£52,000)	106,778 (25.9%)	74,129 (29.5%)	32,649 (20.3%)	
BMI, kg/m²				<0.001***
<18.5	2,071 (0.5%)	1,239 (0.5%)	832 (0.5%)	
≥18.5 to <25.0	134,955 (32.7%)	86,828 (34.5%)	48,127 (30.0%)	
≥25.0 to <30.0	175,962 (42.7%)	107,968 (42.9%)	67,994 (42.3%)	
≥30.0	99,146 (24.1%)	55,539 (22.1%)	43,607 (27.2%)	
Smoking status				<0.001***
never	224,979 (54.6%)	146,190 (58.1%)	78,789 (49.1%)	
past	143,789 (34.9%)	81,576 (32.4%)	62,213 (38.7%)	
current	43,366 (10.5%)	23,808 (9.5%)	19,558 (12.2%)	
Drinking status				<0.001***
never	15,564 (3.8%)	8,596 (3.4%)	6,968 (4.3%)	
past	14,000 (3.4%)	7,260 (2.9%)	6,740 (4.2%)	
current	382,570 (92.8%)	235,718 (93.7%)	146,852 (91.5%)	
Diabetes				<0.001***
No	391,597 (95.0%)	240,910 (95.8%)	150,687 (93.9%)	
Yes	20,537 (5.0%)	10,664 (4.2%)	9,873 (6.1%)	
Hypertension				<0.001***
No	303,357 (73.6%)	190,533 (75.7%)	112,824 (70.3%)	
Yes	108,777 (26.4%)	61,041 (24.3%)	47,736 (29.7%)	
Stroke				<0.001***
No	406,302 (98.6%)	248,770 (98.9%)	157,532 (98.1%)	
Yes	5,832 (1.4%)	2,804 (1.1%)	3,028 (1.9%)	
Oral health problems				/
Mouth ulcers, N (%)	41,770 (10.1%)	/	/	
Painful gums, N (%)	12,204 (3.0%)	/	/	
Bleeding gums, N (%)	55,959 (13.6%)	/	/	
Loose teeth, N (%)	17,481 (4.2%)	/	/	
Toothaches, N (%)	18,325 (4.4%)	/	/	
Dentures, N (%)	64,722 (15.7%)	/	/	
Number of oral problems				/
No oral health problem	251,574 (61.0%)	/	/	
one oral health problem	122,713 (29.8%)	/	/	
two oral health problems	28,804 (7.0%)	/	/	
three oral health problems	6,769 (1.6%)	/	/	
More than three oral problems	2,274 (0.6%)	/	/	

The analysis included all participants and excluded participants who developed CD/UC prior to baseline (N = 412,134). Continuous variables are expressed as mean (standard deviation), and categorical variables are expressed as n (%).

BMI, body mass index. *p<0.05, **p<0.01, ***p<0.001.

### 3.2 The cohort study of association of oral health problems/number of oral health problems and CD/UC

During a median follow-up of 13.8 years, incidence rates of CD and UC per 100,000 person-years among participants were 16.61 and 33.75, with no violations of proportional hazards assumptions observed. We observed that self-reported mouth ulcers (HR: 1.251, 95% CI: 1.025-1.526), painful gums (HR: 1.504, 95% CI: 1.116-2.027), and dentures (HR: 1.298, 95% CI: 1.100-1.531) were linked with increased risk of CD. We did not observe significant associations between bleeding gums, loose teeth, toothache and risk of CD (see **Table 2**).

**Table 2:** Longitudinal association of oral health problems with incident IBD (Models 1 & 2 & 3).

Variable	Incident rate per 100,000 person-years	Model 1		Model 2		Model 3	
		Hazard ratio (95% CI)	p-Value	Hazard ratio (95% CI)	p-Value	Hazard ratio (95% CI)	p-Value
CD							
Oral health problems							
Mouth ulcers	19.70	1.211 (0.993-1.477)	0.0583	1.218 (0.998-1.485)	0.0521	1.251 (1.025-1.526)	0.0276*
Painful gums	28.28	1.740 (1.294-2.341)	0.0003***	1.613 (1.198-2.172)	0.0017**	1.504 (1.116-2.027)	0.0073**
Bleeding gums	18.58	1.139 (0.952-1.363)	0.1550	1.140 (0.951-1.366)	0.1569	1.166 (0.972-1.398)	0.0985
Loose teeth	25.15	1.551 (1.189-2.023)	0.0012**	1.398 (1.070-1.827)	0.0140*	1.225 (0.936-1.603)	0.1400
Toothache	17.07	1.029 (0.755-1.403)	0.8540	0.981 (0.719-1.340)	0.9056	0.935 (0.685-1.277)	0.6726
Dentures	24.71	1.634 (1.401-1.906)	<0.0001***	1.434 (1.217-1.689)	<0.0001***	1.298 (1.100-1.531)	0.0020**
UC							
Oral health problems							
Mouth ulcers	37.31	1.118 (0.969-1.291)	0.1270	1.150 (0.996-1.327)	0.0577	1.162 (1.006-1.342)	0.0409*
Painful gums	38.76	1.153 (0.897-1.483)	0.2660	1.123 (0.873-1.444)	0.3686	1.064 (0.827-1.369)	0.6290
Bleeding gums	32.44	0.955 (0.835-1.092)	0.4990	0.994 (0.868-1.138)	0.9308	0.994 (0.868-1.138)	0.9274
Loose teeth	40.36	1.206 (0.979-1.486)	0.0783	1.091 (0.885-1.345)	0.4142	0.983 (0.796-1.213)	0.8723
Toothache	32.14	0.950 (0.758-1.190)	0.6540	0.922 (0.736-1.157)	0.4866	0.893 (0.712-1.120)	0.3269
Dentures	46.54	1.483 (1.327-1.657)	<0.0001***	1.275 (1.134-1.434)	<0.0001***	1.177 (1.046-1.325)	0.0069**

Data analysed using Cox regression to obtain HR and 95% CI. The analysis included a total of 412,134 participants without CD/UC at baseline, and the model 1 was not adjusted for covariates, the model 2 was adjusted for age, sex, ethnicity, education level, income level, BMI, the model 3 was adjusted for age, sex, ethnicity, education level, income level, BMI, smoking status, drinking status, hypertension, diabetes and stroke status. Incidence rate per 100,000 person-years = (Number of cases with a specific oral health problem/Total person-years with that specific oral health problem) × 100000. Bold text indicates statistically significant associations (<0.05).

HR, Hazard ratio; CI, Confidence interval; BMI, body mass index. *p<0.05, **p<0.01, ***p<0.001.

The results showing the association of oral health problems with the incidence of UC suggested that self-reported mouth ulcers (HR: 1.162, 95% CI: 1.006-1.342) and dentures (HR: 1.177, 95% CI: 1.046-1.325) were significantly associated with increased risk of UC. We did not observe significant associations between painful gums, bleeding gums, loose teeth, toothache and risk of UC (see **Table 2**).

The number of oral health problems has been shown to be associated with CD, but not with UC. Having two (HR: 1.294, 95% CI: 1.025-1.634), three (HR: 1.687, 95% CI: 1.143-2.490) and more than three (HR: 2.128, 95% CI: 1.197-3.781) oral health problems showed a significant correlation with the incidence of CD compared with those without any oral health problems (see **Table 3**), and with the increasing number of oral health problems, the risk of CD rose (P < 0.001 for trend). In addition, we performed imputation for missing covariates, and the results did not change substantially. The sensitivity analyses yielded stable results (see **Supplementary Tables 4-9**).

**Table 3:** Longitudinal association of the number of oral health problems with incident IBD (Models 1 & 2 & 3).

Variable	Model 1			Model 2			Model 3		
	Hazard ratio (95% CI)	p-Value	p-Value for Trend	Hazard ratio (95% CI)	p-Value	p-Value for Trend	Hazard ratio (95% CI)	p-Value	p-Value for Trend
CD									
Number of oral problems									
No oral health problem	1 (reference)		1.42×10^-9^***	1 (reference)		1.39×10-6***	1 (reference)		6.45×10-5***
one oral health problem	1.280 (1.110-1.477)	0.0007***		1.184 (1.025-1.369)	0.02170*		1.133 (0.980-1.310)	0.0908	
two oral health problems	1.505 (1.194-1.897)	0.0005***		1.369 (1.085-1.728)	0.00810**		1.294 (1.025-1.634)	0.0303*	
three oral health problems	2.065 (1.402-3.042)	0.0002***		1.836 (1.245-2.709)	0.00219**		1.687 (1.143-2.490)	0.0085**	
More than three oral problems	2.765 (1.559-4.901)	0.0005***		2.415 (1.360-4.288)	0.00261**		2.128 (1.197-3.781)	0.0100*	
UC									
Number of oral problems									
No oral health problem	1 (reference)		2.86×10-5***	1 (reference)		0.0041**	1 (reference)		0.0528
one oral health problem	1.206 (1.0922-1.332)	0.0002***		1.13 (1.020-1.247)	0.0193*		1.083 (0.979-1.198)	0.1217	
two oral health problems	1.213 (1.0199-1.443)	0.0290*		1.136 (0.954-1.353)	0.1514		1.074 (0.901-1.279)	0.4262	
three oral health problems	1.499 (1.1013-2.041)	0.0101*		1.391 (1.021-1.895)	0.0367*		1.284 (0.942-1.750)	0.1137	
More than three oral problems	1.287 (0.7288-2.274)	0.3842		1.174 (0.664-2.075)	0.5813		1.047 (0.592-1.852)	0.8735	

Data analysed using Cox regression to obtain HR and 95% CI. The analysis included a total of 412,134 participants without CD/UC at baseline, and the model 1 was not adjusted for covariates, the model 2 was adjusted for age, sex, ethnicity, education level, income level, BMI, the model 3 was adjusted for age, sex, ethnicity, education level, income level, BMI, smoking status, drinking status, hypertension, diabetes and stroke status. Bold text indicates statistically significant associations (<0.05).

HR, Hazard ratio; CI, Confidence interval; BMI, body mass index. *p<0.05, **p<0.01, ***p<0.001.

### 3.3 Mediation analysis of inflammatory factors on association of oral health problems and CD/UC

**Figure 2:**
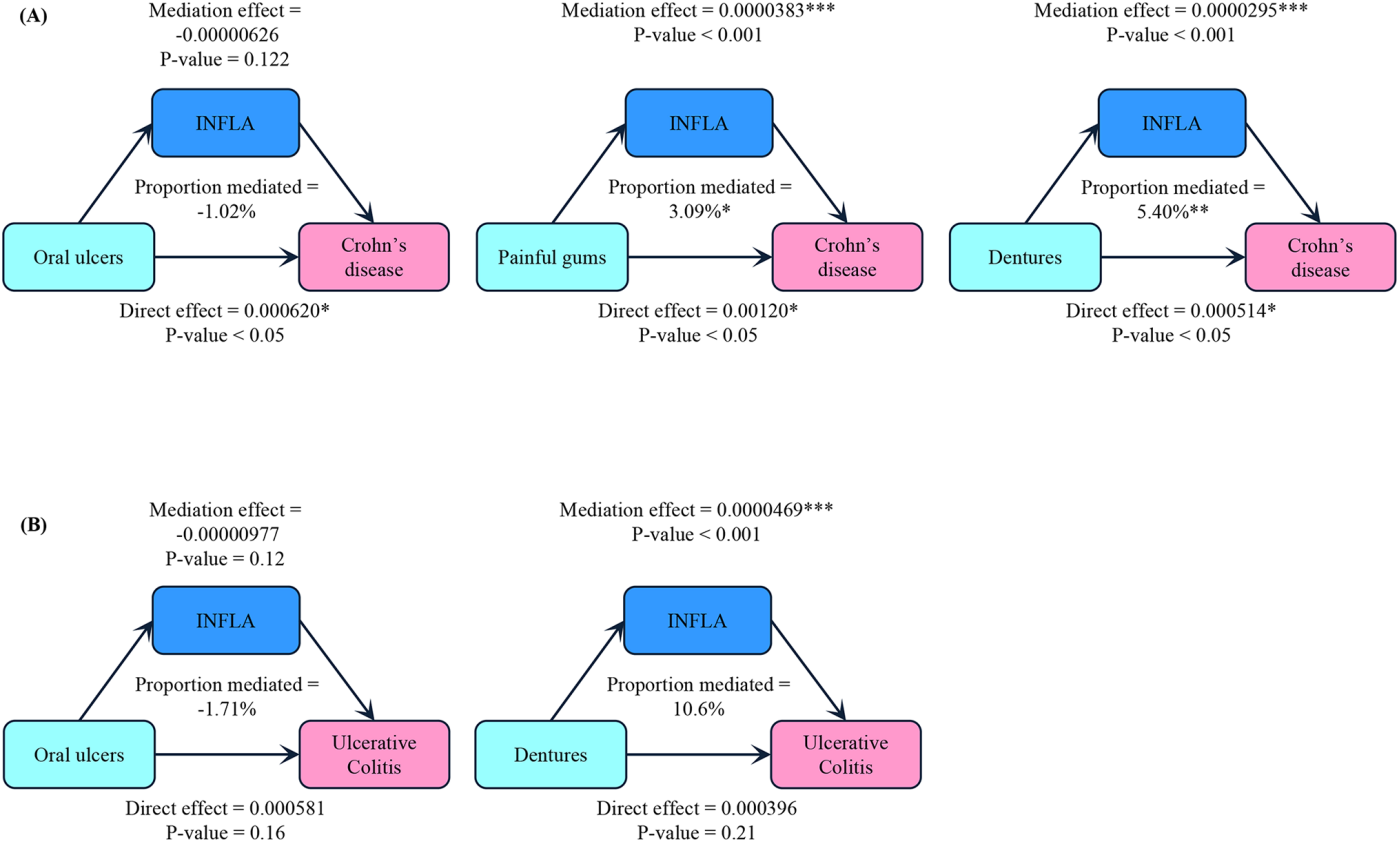
Mediation analysis path diagram. **(A)** Mediation analysis of INFLA on the association between oral health problems and CD. **(B)** Mediation analysis of INFLA on the association between oral health problems and UC. The mediation effect indicated whether INFLA was in the pathway between oral health and CD/UC. The proportion mediated = Indirect effect / [Indirect effect + Direct effect]. Adjusted for age, sex, ethnicity, education level, income level, BMI, smoking status, drinking status, hypertension, diabetes and stroke status. INFLA, low-grade inflammation index. BMI, body mass index.

A mediation analysis was utilized to explore the mediating role of inflammatory factors (see **Figure 2**), and shows the mediating role of inflammatory factors on association of oral health problems and CD. As a mediating variable, INFLA demonstrated a mediating role in the association between oral health problems and CD, with mediation proportions of 3.09% for painful gums and 5.40% for dentures (p < 0.05). We found no evidence of INFLA mediating the association between mouth ulcers and CD.

In addition, our analysis revealed no significant mediating role of INFLA on the associations between mouth ulcers/dentures and UC.

### 3.4 The subgroup analysis of association of oral health problems and CD/UC

**Figure 3:**
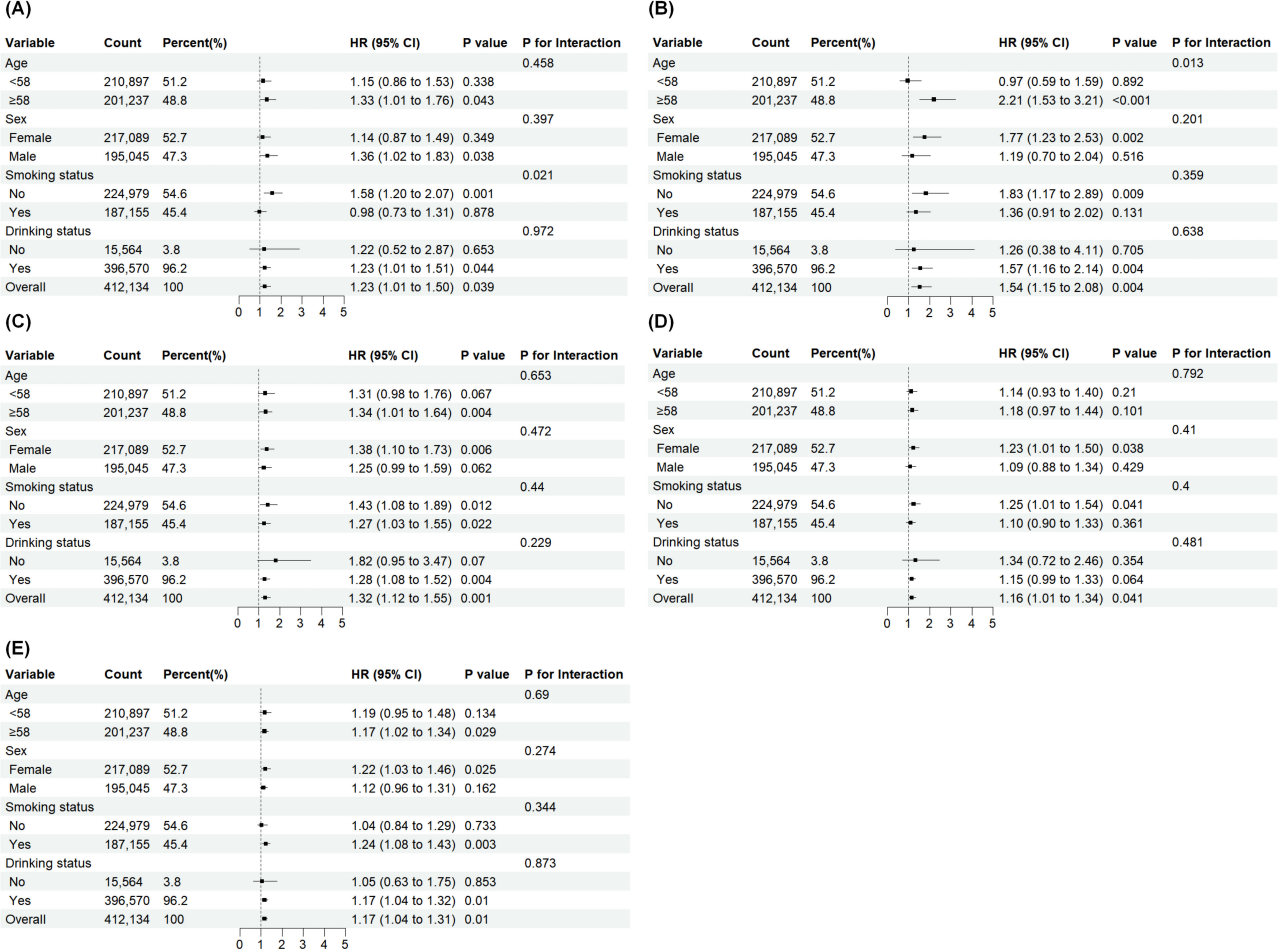
Subgroup analysis of the association between oral health problems and IBD. Subgroup analysis of the association between **(A)** mouth ulcers with incident Crohn’s disease **(B)** painful gums with incident Crohn’s disease **(C)** dentures with incident Crohn’s disease **(D)** mouth ulcers with incident ulcerative colitis **(E)** dentures with incident ulcerative colitis. All analyses were performed using Cox regression with models adjusted for baseline age, sex, ethnicity, education level, income level, BMI, smoking status, drinking status, hypertension, diabetes and stroke status. HR, Hazard ratio; CI, Confidence interval. BMI, body mass index. Likelihood ratio tests were applied to formally test for interactions.

Subgroup analysis was utilized to evaluate the comparative risk of CD/UC by age, sex, smoking status and drinking status (see **Figure 3**). Participants were stratified by median age (58 years) into the <58 and ≥58 years subgroups to compare the risk of CD between the oral health problems group and the reference group. In the association with CD, we found that mouth ulcers, painful gums and dentures were associated with increased risk of CD among individuals aged ≥58 years, non-smokers and drinkers. Sex-stratified analyses indicated that painful gums and dentures might increase risk of CD in females, while oral ulcers were specifically associated with increased risk of CD among male participants.

Furthermore, we found that mouth ulcers increase risk of UC onset in female and non-smokers, while dentures elevate risk of UC in populations aged ≥58 years, female, smokers and drinkers.

## 4 Discussion

This large UK cohort study reported that mouth ulcers and dentures increased the risk of CD and UC. Painful gums was associated with CD, but not with UC. However, we did not observe an association between bleeding gums and loose teeth with CD. Notably, the increase in CD risk with accumulating oral health problems suggested that cumulative oral health burden may exert a pronounced impact on risk of CD. In subgroup analyses, higher risk estimates were observed among non-smokers. Previous studies have suggested that smoking may exert a protective effect against UC. This protective effect may be attributed to the upregulation of the mucosal homing receptor GPR15, which promotes anti-inflammatory responses of Treg cells (32). Furthermore, smoking is a strong risk factor for CD, and the elevated baseline risk among smokers may mask the potential effect of oral health problems on CD. It must be emphasized that these findings do not support a protective role for smoking in IBD, as its overall harm far outweighs any hypothetical and unsubstantiated local effects. In mediation analyses, inflammatory factors were found to mediate the association between these oral health problems and CD, the positive mediating role of INFLA in the association between painful gums/dentures and CD was pronounced, indicating that inflammatory responses might have a potential role in this pathway. While no inflammatory mediating role was observed between oral health problems and UC, mediation analysis revealed a significant indirect effect of INFLA in the relationship between denture and UC, with no significant direct effect. This suggests that INFLA may fully mediate this association, indicating that dentures likely influence UC primarily through inflammatory pathways. The mediating role of INFLA on the relationship between oral health problems and CD was relatively low, suggesting that other potential mechanisms may be involved.

Accumulating evidence has highlighted the association between oral health problems and IBD. For the first time, we demonstrated through a prospective cohort study that more detailed assessment covering oral health problems increase the risk of IBD, consistent with previous cross-sectional and case-control studies. For example, Kato, Ikuko et al. and Mejia-Lancheros, Cilia et al. showed that oral health problems are risk factors for IBD (18, 19). Contrary to our data, findings from a Swedish cohort indicated that oral health problems exerted a protective influence on IBD, the specific mechanisms underlying this requires further investigation in future studies (20). Our study found no significant association between bleeding gums/loose teeth and CD, this may be because bleeding gums are more frequently caused by trauma than by pathological factors and there is no report on whether bleeding gums is caused by trauma in the UK Biobank. Additionally, self-reported loose teeth is highly subjective, and bias may occur due to the lack of professional evaluation. Finally, growing attention has been paid to the association between periapical conditions and IBD in recent studies, with research by La Rosa et al. and Wang et al. suggesting a potential link between periapical conditions and IBD (33, 34). As periapical conditions were not included in our study, future research could expand the scope of oral health problems assessed.

Oral health problems may increase IBD risk through several potential mechanisms. Firstly, the pathogenesis of IBD may be influenced by biological mechanisms whereby periodontal lesions induced inflammatory responses, which can occur through increasing and spreading serum inflammatory factors into the systemic circulation, thus increasing the systemic inflammatory burden. Moreover, oral pathogenic bacteria may translocate to the gut, where colonization can induce immune activation. Following intestinal epithelial damage, these bacteria engage gut-resident immune cells, stimulating IBD occurs (35). Our mediation analysis supported further evidence that INFLA-score positively mediated the association between several oral health and CD. Finally, a large number of oral pathogens produced by oral health problems can also activate Th17 cells, which have intestinal tropism and cause IBD (36).

The study possesses several strengths. First, our results were derived from a large population-based cohort, with relevant confounding factors and covariates adjusted for, which strengthen the ability in causal inference verification. Second, the classification of oral health problems in our study is more detailed and covers major issues, which can better reveal oral health problems increased the risk of IBD. Third, our study is the first to demonstrate that the incidence of CD increases significantly as number of oral health problems rises. Finally, we evaluated the mediating role and effect magnitude of inflammatory factors in the associations between oral health problems and CD.

Several limitations should be noted. First, oral health status could change over the process of follow-up, and we lacked data on oral problems severity, duration, or treatment status. This may reduce the clinical specificity of the exposure. Second, we used self-reported measures based on prior studies, which may lead to the misclassification and reporting bias, particularly for conditions such as bleeding gums or loose teeth. Third, despite adjustment for various covariates, potential residual confounding may still exist due to a lack of data on factors such as oral hygiene behaviors. Fourth, inflammatory markers were measured only at baseline, which may not adequately represent inflammatory processes over the entire follow-up period. Fifth, incident IBD was identified from multiple sources, with cases defined by any single diagnostic record. This approach may reduce specificity, risking false positives. Finally, the participants from UK Biobank primarily comprises White and generally healthier than the general population, which may not fully generalize across other ethnic groups.

## 5 Conclusion

Mouth ulcers and dentures might increase the risk of CD and UC. Painful gums was associated with CD, but not UC. Further, patients with a greater number of oral health problems are more likely to develop CD. In view of the positive correlation between oral health problems and the risk of IBD, we recommend strengthening the supervision of the occurrence of various oral health problems to reduce the occurrence of IBD. This study also suggested the mediating role of inflammatory factors in this process, but further underlying mechanisms in the inflammatory association are still needed. From the perspective of public health, reducing the occurrence of oral health problems is of great significance for maintaining intestinal health and preventing intestinal diseases. In clinical practice, active oral health surveillance and oral care should be implemented, as improving oral health may serve as a key strategy to reduce the incidence of IBD.

## Data Availability

Publicly available datasets were analyzed in this study. This data can be found here: https://www.ukbiobank.ac.uk.
