# Supplementary material for: Associations between oral health problems and inflammatory bowel disease: evidence from prospective cohort study
Source: Front Immunol. 2026 Mar 12;17:1751956. doi: 10.3389/fimmu.2026.1751956 (PMC13018159; doi:10.3389/fimmu.2026.1751956)
Supplement: Supplementary file 1 [file DataSheet1.docx]

***Supplementary Material: Associations between oral health problems and inflammatory bowel disease: evidence from prospective cohort study***

**Yixiao Wang**, wyx0124@163.com; Tel: +8653282991712; Fax: +8653283801449

Qingdao Stomatological Hospital Affiliated to Qingdao University, Qingdao, China; School of Public Health, Qingdao University, Qingdao, China.

**Xiuxiu Sun**, 13791224384@163.com; Tel: +8653282991712; Fax: +8653283801449

School of Public Health, Qingdao University, Qingdao, China.

**Jiahao Chen**, chenjh1208@126.com.

Qingdao Stomatological Hospital Affiliated to Qingdao University, Qingdao, China.

**Tong Wang**, wangtong4@qdu.edu.cn; Tel: +8653282991712; Fax: +8653283801449

School of Public Health, Qingdao University, Qingdao, China.

**Corresponding author:**

**Wanchun Wang***, wangwanchun2019@qdu.edu.cn.

Qingdao Stomatological Hospital Affiliated to Qingdao University, Qingdao, China.

**Dongfeng Zhang***, zhangdf1961@126.com; Tel: +8653282991712; Fax: +8653283801449

School of Public Health, Qingdao University, Qingdao, China.

**Supplementary File**

[Supplementary Table 1. Baseline characteristics of participants according to presence of oral health problems. 3](#_Toc8550)

[Supplementary Table 2. Longitudinal association of oral health problems with incident IBD (Models 1 & 2 & 3). 5](#_Toc21506)

[Supplementary Table 3. Longitudinal association of the number of oral health problems with incident IBD (Models 1 & 2 & 3). 7](#_Toc8273)

[Supplementary Table 4. Sensitivity analysis of oral health problems with incident IBD: participants with ≤2 years of follow-up were excluded. 9](#_Toc8558)

[Supplementary Table 5. Sensitivity analysis of the number of oral health problems with incident IBD : participants with ≤2 years of follow-up were excluded. 10](#_Toc29865)

[Supplementary Table 6. Sensitivity analysis of oral health problems with incident IBD : participants with colorectal cancer were excluded. 10](#_Toc7478)

[Supplementary Table 7. Sensitivity analysis of the number of oral health problems with incident IBD : participants with colorectal cancer were excluded. 11](#_Toc2822)

[Supplementary Table 8. Sensitivity analysis of oral health problems with incident IBD : multiple imputation for missing covariates. 12](#_Toc24738)

[Supplementary Table 9. Sensitivity analysis of the number of oral health problems with incident IBD : multiple imputation for missing covariates. 13](#_Toc16986)

[Supplementary Table 10. Mediation analysis of INFLA in the association of oral health problems and Crohn’s disease. 13](#_Toc30335)

[Supplementary Table 11. Mediation analysis of INFLA in the association of oral health problems and ulcerative colitis. 14](#_Toc2902)

[Supplementary Figure 1. Mediation analysis path diagram. 15](#_Toc16748)

[Supplementary Figure 2. Subgroup analysis of the association between mouth ulcers with incident Crohn’s disease. 16](#_Toc26636)

[Supplementary Figure 3. Subgroup analysis of the association between painful gums with incident Crohn’s disease. 17](#_Toc25973)

[Supplementary Figure 4. Subgroup analysis of the association between dentures with incident Crohn’s disease. 18](#_Toc30095)

[Supplementary Figure 5. Subgroup analysis of the association between mouth ulcers with incident ulcerative colitis. 19](#_Toc22451)

[Supplementary Figure 6. Subgroup analysis of the association between dentures with incident ulcerative colitis. 20](#_Toc14238)

[Supplementary Methods 21](#_Toc3021)

**Supplementary Table 1. Baseline characteristics of participants according to presence of oral health problems.**

| **Characteristic** | **All**  **(N = 412,134)** | **Oral health problems (-)**  **(N = 251,574)** | **Oral health problems (+)**  **(N =160,560)** | **p-Value** |
| --- | --- | --- | --- | --- |
| Age at baseline, years |  |  |  | **<0.001***** |
| <45 | 44,847 (10.9%) | 29,773 (11.8%) | 15,074 (9.4%) |  |
| 45-54 | 122,301 (29.7%) | 79,521 (31.6%) | 42,780 (26.6%) |  |
| 55-64 | 172,517 (41.9%) | 104,034 (41.4%) | 68,483 (42.7%) |  |
| ≥65 | 72,469 (17.6%) | 38,246 (15.2%) | 34,223 (21.3%) |  |
| Sex |  |  |  | **<0.001***** |
| Female | 217,089 (52.7%) | 130,442 (51.9%) | 86,647 (54.0%) |  |
| Male | 195,045 (47.3%) | 121,132 (48.1%) | 73,913 (46.0%) |  |
| Ethnicity |  |  |  | **<0.001***** |
| White | 393,010 (95.4%) | 241,021 (95.8%) | 151,989 (94.7%) |  |
| Mixed | 5,498 (1.3%) | 3,208 (1.3%) | 2,290 (1.4%) |  |
| Asian or Asian British | 7,792 (1.9%) | 4,221 (1.7%) | 3,571 (2.2%) |  |
| Black or Black British | 5,834 (1.4%) | 3,124 (1.2%) | 2,710 (1.7%) |  |
| Education |  |  |  | **<0.001***** |
| College or University degree | 144,794 (35.1%) | 96,655 (38.4%) | 48,139 (30.0%) |  |
| Others | 267,340 (64.9%) | 154,919 (61.6%) | 112,421 (70.0%) |  |
| Income levels |  |  |  | **<0.001***** |
| Level 1 (<£18000) | 92,527 (22.5%) | 46,182 (18.4%) | 46,345 (28.9%) |  |
| Level 2 (£8000–30999) | 104,833 (25.4%) | 61,686 (24.5%) | 43,147 (26.9%) |  |
| Level 3 (£31000–52000) | 107,996 (26.2%) | 69,577 (27.7%) | 38,419 (23.9%) |  |
| Level 4 (>£52000) | 106,778 (25.9%) | 74,129 (29.5%) | 32,649 (20.3%) |  |
| BMI, kg/m² |  |  |  | **<0.001***** |
| <18.5 | 2,071 (0.5%) | 1,239 (0.5%) | 832 (0.5%) |  |
| ≥18.5 to <25.0 | 134,955 (32.7%) | 86,828 (34.5%) | 48,127 (30.0%) |  |
| ≥25.0 to <30.0 | 175,962 (42.7%) | 107,968 (42.9%) | 67,994 (42.3%) |  |
| ≥30.0 | 99,146 (24.1%) | 55,539 (22.1%) | 43,607 (27.2%) |  |
| Smoking status |  |  |  | **<0.001***** |
| never | 224,979 (54.6%) | 146,190 (58.1%) | 78,789 (49.1%) |  |
| past | 143,789 (34.9%) | 81,576 (32.4%) | 62,213 (38.7%) |  |
| current | 43,366 (10.5%) | 23,808 (9.5%) | 19,558 (12.2%) |  |
| Drinking status |  |  |  | **<0.001***** |
| never | 15,564 (3.8%) | 8,596 (3.4%) | 6,968 (4.3%) |  |
| past | 14,000 (3.4%) | 7,260 (2.9%) | 6,740 (4.2%) |  |
| current | 382,570 (92.8%) | 235,718 (93.7%) | 146,852 (91.5%) |  |
| Diabetes |  |  |  | **<0.001***** |
| No | 391,597 (95.0%) | 240,910 (95.8%) | 150,687 (93.9%) |  |
| Yes | 20,537 (5.0%) | 10,664 (4.2%) | 9,873 (6.1%) |  |
| Hypertension |  |  |  | **<0.001***** |
| No | 303,357 (73.6%) | 190,533 (75.7%) | 112,824 (70.3%) |  |
| Yes | 108,777 (26.4%) | 61,041 (24.3%) | 47,736 (29.7%) |  |
| Stroke |  |  |  | **<0.001***** |
| No | 406,302 (98.6%) | 248,770 (98.9%) | 157,532 (98.1%) |  |
| Yes | 5,832 (1.4%) | 2,804 (1.1%) | 3,028 (1.9%) |  |
| Oral health problems |  |  |  | / |
| Mouth ulcers, N (%) | 41,770 (10.1%) | / | / |  |
| Painful gums, N (%) | 12,204 (3.0%) | / | / |  |
| Bleeding gums, N (%) | 55,959 (13.6%) | / | / |  |
| Loose teeth, N (%) | 17,481 (4.2%) | / | / |  |
| Toothaches, N (%) | 18,325 (4.4%) | / | / |  |
| Dentures, N (%) | 64,722 (15.7%) | / | / |  |
| Number of oral problems |  |  |  | / |
| No oral health problem | 251,574 (61.0%) | / | / |  |
| one oral health problem | 122,713 (29.8%) | / | / |  |
| two oral health problems | 28,804 (7.0%) | / | / |  |
| three oral health problems | 6,769 (1.6%) | / | / |  |
| More than three oral problems | 2,274 (0.6%) | / | / |  |

***Note:*** The analysis included all participants and excluded participants who developed CD/UC prior to baseline (N = 412,134). Continuous variables are expressed as mean (standard deviation), and categorical variables are expressed as n (%).

Abbreviation: BMI: body mass index. *p<0.05, **p<0.01, ***p<0.001.

**Supplementary Table 2. Longitudinal association of oral health problems with incident IBD (Models 1 & 2 & 3)**.

**I. Model 1**

| **Variable** | **Incident Crohn’s disease** | | | **Incident ulcerative colitis** | | |
| --- | --- | --- | --- | --- | --- | --- |
|  | **Incident rate per 100,000 person-years** | **Hazard ratio (95% CI)** | **p-Value** | **Incident rate per 100,000 person-years** | **Hazard ratio (95% CI)** | **p-Value** |
| **Oral health problems** | | | | | | |
| Mouth ulcers | 19.70 | 1.211 (0.993-1.477) | 0.0583 | 37.31 | 1.118 (0.969-1.291) | 0.1270 |
| Painful gums | 28.28 | 1.740 (1.294-2.341) | **0.0003***** | 38.76 | 1.153 (0.897-1.483) | 0.2660 |
| Bleeding gums | 18.58 | 1.139 (0.952-1.363) | 0.1550 | 32.44 | 0.955 (0.835-1.092) | 0.4990 |
| Loose teeth | 25.15 | 1.551 (1.189-2.023) | **0.0012**** | 40.36 | 1.206 (0.979-1.486) | 0.0783 |
| Toothache | 17.07 | 1.029 (0.755-1.403) | 0.8540 | 32.14 | 0.950 (0.758-1.190) | 0.6540 |
| Dentures | 24.71 | 1.634 (1.401-1.906) | **<0.0001***** | 46.54 | 1.483 (1.327-1.657) | **<0.0001***** |

***Note:*** Data analysed using Cox regression to obtain HR and 95% CI. The analysis included a total of 412,134 participants without CD/UC at baseline, and the model was not adjusted for covariates. Incidence rate per 100,000 person-years = (Number of cases with a specific oral health problem / Total person-years with that specific oral health problem) × 100000. Bold text indicates statistically significant associations (<0.05).

Abbreviation: HR: Hazard ratio, CI: Confidence interval. *p<0.05, **p<0.01, ***p<0.001.

**I. Model 2**

| **Variable** | **Incident Crohn’s disease** | | | **Incident ulcerative colitis** | | |
| --- | --- | --- | --- | --- | --- | --- |
|  | **Incident rate per 100,000 person-years** | **Hazard ratio (95% CI)** | **p-Value** | **Incident rate per 100,000 person-years** | **Hazard ratio (95% CI)** | **p-Value** |
| **Oral health problems** | | | | | | |
| Mouth ulcers | 19.70 | 1.218 (0.998-1.485) | 0.0521 | 37.31 | 1.150 (0.996-1.327) | 0.0577 |
| Painful gums | 28.28 | 1.613 (1.198-2.172) | **0.0017**** | 38.76 | 1.123 (0.873-1.444) | 0.3686 |
| Bleeding gums | 18.58 | 1.140 (0.951-1.366) | 0.1569 | 32.44 | 0.994 (0.868-1.138) | 0.9308 |
| Loose teeth | 25.15 | 1.398 (1.070-1.827) | **0.0140*** | 40.36 | 1.091 (0.885-1.345) | 0.4142 |
| Toothache | 17.07 | 0.981 (0.719-1.340) | 0.9056 | 32.14 | 0.922 (0.736-1.157) | 0.4866 |
| Dentures | 24.71 | 1.434 (1.217-1.689) | **<0.0001***** | 46.54 | 1.275 (1.134-1.434) | **<0.0001***** |

***Note:*** Data analysed using Cox regression to obtain HR and 95% CI. The analysis included a total of 412,134 participants without CD/UC at baseline, and the model was adjusted for age, sex, ethnicity, education level, income level, BMI. Incidence rate per 100,000 person-years = (Number of cases with a specific oral health problem / Total person-years with that specific oral health problem) × 100000. Bold text indicates statistically significant associations (<0.05).

Abbreviation: HR: Hazard ratio, CI: Confidence interval. BMI: body mass index. *p<0.05, **p<0.01, ***p<0.001.

**I. Model 3**

| **Variable** | **Incident Crohn’s disease** | | | **Incident ulcerative colitis** | | |
| --- | --- | --- | --- | --- | --- | --- |
|  | **Incident rate per 100,000 person-years** | **Hazard ratio (95% CI)** | **p-Value** | **Incident rate per 100,000 person-years** | **Hazard ratio (95% CI)** | **p-Value** |
| **Oral health problems** | | | | | | |
| Mouth ulcers | 19.70 | 1.251 (1.025-1.526) | **0.0276*** | 37.31 | 1.162 (1.006-1.342) | **0.0409*** |
| Painful gums | 28.28 | 1.504 (1.116-2.027) | **0.0073**** | 38.76 | 1.064 (0.827-1.369) | 0.6290 |
| Bleeding gums | 18.58 | 1.166 (0.972-1.398) | 0.0985 | 32.44 | 0.994 (0.868-1.138) | 0.9274 |
| Loose teeth | 25.15 | 1.225 (0.936-1.603) | 0.1400 | 40.36 | 0.983 (0.796-1.213) | 0.8723 |
| Toothache | 17.07 | 0.935 (0.685-1.277) | 0.6726 | 32.14 | 0.893 (0.712-1.120) | 0.3269 |
| Dentures | 24.71 | 1.298 (1.100-1.531) | **0.0020**** | 46.54 | 1.177 (1.046-1.325) | **0.0069**** |

***Note:*** Data analysed using Cox regression to obtain HR and 95% CI. The analysis included a total of 412,134 participants without CD/UC at baseline, and the model was adjusted for age, sex, ethnicity, education level, income level, BMI, smoking status, drinking status, hypertension, diabetes and stroke status. Incidence rate per 100,000 person-years = (Number of cases with a specific oral health problem / Total person-years with that specific oral health problem) × 100000. Bold text indicates statistically significant associations (<0.05).

Abbreviation: HR: Hazard ratio, CI: Confidence interval. BMI: body mass index. *p<0.05, **p<0.01, ***p<0.001.

**Supplementary Table 3. Longitudinal association of the number of oral health problems with incident IBD (Models 1 & 2 & 3)**.

**I. Model 1**

| **Variable** | **Incident Crohn’s disease** | | | **Incident ulcerative colitis** | | |
| --- | --- | --- | --- | --- | --- | --- |
|  | **Hazard ratio (95%CI)** | **p-Value** | **p-Value for Trend** | **Hazard ratio (95%CI)** | **p-Value** | **p-Value for Trend** |
| **Number of oral problems** |  |  |  |  |  |  |
| No oral health problem | 1 (reference) |  | **1.42×10^-9^***** | 1 (reference) |  | **2.86×10^-5^***** |
| one oral health problem | 1.280 (1.110-1.477) | **0.0007***** |  | 1.206 (1.0922-1.332) | **0.0002***** |  |
| two oral health problems | 1.505 (1.194-1.897) | **0.0005***** |  | 1.213 (1.0199-1.443) | **0.0290*** |  |
| three oral health problems | 2.065 (1.402-3.042) | **0.0002***** |  | 1.499 (1.1013-2.041) | **0.0101*** |  |
| More than three oral problems | 2.765 (1.559-4.901) | **0.0005***** |  | 1.287 (0.7288-2.274) | 0.3842 |  |

***Note:*** Data analysed using Cox regression to obtain HR and 95% CI. The analysis included a total of 412,134 participants without CD/UC at baseline, and the model was not adjusted for covariates. Bold text indicates statistically significant associations (<0.05).

Abbreviation: HR: Hazard ratio, CI: Confidence interval. *p<0.05, **p<0.01, ***p<0.001.

**I. Model 2**

| **Variable** | **Incident Crohn’s disease** | | | **Incident ulcerative colitis** | | |
| --- | --- | --- | --- | --- | --- | --- |
|  | **Hazard ratio (95%CI)** | **p-Value** | **p-Value for Trend** | **Hazard ratio (95%CI)** | **p-Value** | **p-Value for Trend** |
| **Number of oral problems** |  |  |  |  |  |  |
| No oral health problem | 1 (reference) |  | **1.39×10^-6^***** | 1 (reference) |  | **0.0041**** |
| one oral health problem | 1.184 (1.025-1.369) | **0.02170*** |  | 1.13 (1.020-1.247) | **0.0193*** |  |
| two oral health problems | 1.369 (1.085-1.728) | **0.00810**** |  | 1.136 (0.954-1.353) | 0.1514 |  |
| three oral health problems | 1.836 (1.245-2.709) | **0.00219**** |  | 1.391 (1.021-1.895) | **0.0367*** |  |
| More than three oral problems | 2.415 (1.360-4.288) | **0.00261**** |  | 1.174 (0.664-2.075) | 0.5813 |  |

***Note:*** Data analysed using Cox regression to obtain HR and 95% CI. The analysis included a total of 412,134 participants without CD/UC at baseline, and the model was adjusted for age, sex, ethnicity, education level, income level, BMI. Bold text indicates statistically significant associations (<0.05).

Abbreviation: HR: Hazard ratio, CI: Confidence interval. BMI: body mass index. *p<0.05, **p<0.01, ***p<0.001.

**I. Model 3**

| **Variable** | **Incident Crohn’s disease** | | | **Incident ulcerative colitis** | | |
| --- | --- | --- | --- | --- | --- | --- |
|  | **Hazard ratio (95%CI)** | **p-Value** | **p-Value for Trend** | **Hazard ratio (95%CI)** | **p-Value** | **p-Value for Trend** |
| **Number of oral problems** |  |  |  |  |  |  |
| No oral health problem | 1 (reference) |  | **6.45×10^-5^***** | 1 (reference) |  | 0.0528 |
| one oral health problem | 1.133 (0.980-1.310) | 0.0908 |  | 1.083 (0.979-1.198) | 0.1217 |  |
| two oral health problems | 1.294 (1.025-1.634) | **0.0303*** |  | 1.074 (0.901-1.279) | 0.4262 |  |
| three oral health problems | 1.687 (1.143-2.490) | **0.0085**** |  | 1.284 (0.942-1.750) | 0.1137 |  |
| More than three oral problems | 2.128 (1.197-3.781) | **0.0100*** |  | 1.047 (0.592-1.852) | 0.8735 |  |

***Note:*** Data analysed using Cox regression to obtain HR and 95% CI. The analysis included a total of 412,134 participants without CD/UC at baseline, and the model was adjusted for age, sex, ethnicity, education level, income level, BMI, smoking status, drinking status, hypertension, diabetes and stroke status. Bold text indicates statistically significant associations (<0.05).

Abbreviation: HR: Hazard ratio, CI: Confidence interval. BMI: body mass index. *p<0.05, **p<0.01, ***p<0.001.

**Supplementary Table 4. Sensitivity analysis of oral health problems with incident IBD: participants with ≤2 years of follow-up were excluded.**

| **Variable** | **Incident Crohn’s disease** | | | **Incident ulcerative colitis** | | |
| --- | --- | --- | --- | --- | --- | --- |
|  | **Incident rate per 100,000 person-years** | **Hazard ratio (95%CI)** | **p-Value** | **Incident rate per 100,000 person-years** | **Hazard ratio (95%CI)** | **p-Value** |
| **Oral health problems** | | | | | | |
| Mouth ulcers | 17.58 | 1.289 (1.043-1.592) | **0.0188*** | 31.28 | 1.127(0.963-1.318) | 0.1359 |
| Painful gums | 23.38 | 1.436 (1.035-1.993) | **0.0304*** | 32.63 | 1.031(0.783-1.357) | 0.8298 |
| Bleeding gums | 16.35 | 1.178 (0.971-1.431) | 0.0973 | 27.83 | 0.986(0.852-1.142) | 0.8516 |
| Loose teeth | 21.69 | 1.228 (0.919-1.641) | 0.1643 | 36.04 | 1.008(0.807-1.260) | 0.9437 |
| Toothache | 15.05 | 0.959 (0.688-1.336) | 0.8038 | 29.21 | 0.943(0.744-1.196) | 0.6286 |
| Dentures | 21.19 | 1.297 (1.085-1.550) | **0.0043**** | 41.14 | 1.207(1.064-1.370) | **0.0035**** |

***Note:*** This analysis excluded participants with ≤2 years of follow-up. Data analysed using Cox regression to obtain HR and 95% CI. Model adjusted for age, sex, ethnicity, education level, income level, BMI, smoking status, drinking status, hypertension, diabetes and stroke status. Incidence rate per 100,000 person-years = (Number of cases with a specific oral health problem / Total person-years with that specific oral health problem) × 100000. Bold text indicates statistically significant associations (<0.05).

Abbreviation: HR: Hazard ratio, CI: Confidence interval. BMI: body mass index. *p<0.05, **p<0.01, ***p<0.001.

**Supplementary Table 5. Sensitivity analysis of the number of oral health problems with incident IBD : participants with ≤2 years of follow-up were excluded.**

| **Variable** | **Incident Crohn’s disease** | | | **Incident ulcerative colitis** | | |
| --- | --- | --- | --- | --- | --- | --- |
|  | **Hazard ratio (95%CI)** | **p-Value** | **p-Value for Trend** | **Hazard ratio (95%CI)** | **p-Value** | **p-Value for Trend** |
| **Number of oral problems** |  |  |  |  |  |  |
| No oral health problem | 1 (reference) |  | **0.0002***** | 1 (reference) |  | 0.0729 |
| one oral health problem | 1.142 (0.977-1.335) | 0.0947 |  | 1.100(0.987-1.226) | 0.0852 |  |
| two oral health problems | 1.308 (1.019-1.680) | **0.0355*** |  | 1.111(0.923-1.338) | 0.2652 |  |
| three oral health problems | 1.966 (1.329-2.907) | **0.0007***** |  | 1.315(0.945-1.829) | 0.1040 |  |
| More than three oral problems | 1.659 (0.823-3.346) | 0.1572 |  | 0.814(0.405-1.634) | 0.5619 |  |

***Note:*** This analysis excluded participants with ≤2 years of follow-up. Data analysed using Cox regression to obtain HR and 95% CI. Model adjusted for age, sex, ethnicity, education level, income level, BMI, smoking status, drinking status, hypertension, diabetes and stroke status. Bold text indicates statistically significant associations (<0.05).

Abbreviation: HR: Hazard ratio, CI: Confidence interval. BMI: body mass index. *p<0.05, **p<0.01, ***p<0.001.

**Supplementary Table 6. Sensitivity analysis of oral health problems with incident IBD : participants with colorectal cancer were excluded.**

| **Variable** | **Incident Crohn’s disease** | | | **Incident ulcerative colitis** | | |
| --- | --- | --- | --- | --- | --- | --- |
|  | **Incident rate per 100,000 person-years** | **Hazard ratio (95%CI)** | **p-Value** | **Incident rate per 100,000 person-years** | **Hazard ratio (95%CI)** | **p-Value** |
| **Oral health problems** | | | | | | |
| Mouth ulcers | 19.11 | 1.239 (1.011-1.518) | **0.0393*** | 36.45 | 1.164 (1.005-1.348) | **0.0428*** |
| Painful gums | 28.74 | 1.559 (1.156-2.101) | **0.0036**** | 37.52 | 1.053 (0.814-1.364) | 0.6938 |
| Bleeding gums | 18.05 | 1.151 (0.956-1.386) | 0.1367 | 31.44 | 0.986 (0.859-1.133) | 0.8450 |
| Loose teeth | 23.92 | 1.178 (0.892-1.556) | 0.2487 | 39.45 | 0.985 (0.794-1.221) | 0.8910 |
| Toothache | 16.51 | 0.918 (0.668-1.264) | 0.6010 | 32.64 | 0.934 (0.745-1.172) | 0.5568 |
| Dentures | 24.12 | 1.286 (1.086-1.523) | **0.0036**** | 45.88 | 1.188 (1.053-1.341) | **0.0051**** |

***Note:*** This analysis excluded participants with colorectal cancer of follow-up. Data analysed using Cox regression to obtain HR and 95% CI. Model adjusted for age, sex, ethnicity, education level, income level, BMI, smoking status, drinking status, hypertension, diabetes and stroke status. Incidence rate per 100,000 person-years = (Number of cases with a specific oral health problem / Total person-years with that specific oral health problem) × 100000. Bold text indicates statistically significant associations (<0.05).

Abbreviation: HR: Hazard ratio, CI: Confidence interval. BMI: body mass index. *p<0.05, **p<0.01, ***p<0.001.

**Supplementary Table 7. Sensitivity analysis of the number of oral health problems with incident IBD : participants with colorectal cancer were excluded.**

| **Variable** | **Incident Crohn’s disease** | | | **Incident ulcerative colitis** | | |
| --- | --- | --- | --- | --- | --- | --- |
|  | **Hazard ratio (95%CI)** | **p-Value** | **p-Value for Trend** | **Hazard ratio (95%CI)** | **p-Value** | **p-Value for Trend** |
| **Number of oral problems** |  |  |  |  |  |  |
| No oral health problem | 1 (reference) |  | **6.45×10^-5^***** | 1 (reference) |  | 0.0602 |
| one oral health problem | 1.132 (0.976-1.312) | 0.1006 |  | 1.093 (0.986-1.212) | 0.0895 |  |
| two oral health problems | 1.293 (1.020-1.639) | **0.0341*** |  | 1.063 (0.888-1.272) | 0.5073 |  |
| three oral health problems | 1.610 (1.075-2.412) | **0.0210*** |  | 1.308 (0.956-1.789) | 0.0937 |  |
| More than three oral problems | 2.014 (1.106-3.670) | **0.0222*** |  | 1.096 (0.620-1.939) | 0.7524 |  |

***Note:*** This analysis excluded participants with colorectal cancer of follow-up. Data analysed using Cox regression to obtain HR and 95% CI. Model adjusted for age, sex, ethnicity, education level, income level, BMI, smoking status, drinking status, hypertension, diabetes and stroke status. Bold text indicates statistically significant associations (<0.05).

Abbreviation: HR: Hazard ratio, CI: Confidence interval. BMI: body mass index. *p<0.05, **p<0.01, ***p<0.001.

**Supplementary Table 8. Sensitivity analysis of oral health problems with incident IBD : multiple imputation for missing covariates.**

| **Variable** | **Incident Crohn’s disease** | | | **Incident ulcerative colitis** | | |
| --- | --- | --- | --- | --- | --- | --- |
|  | **Incident rate per 100,000 person-years** | **Hazard ratio (95%CI)** | **p-Value** | **Incident rate per 100,000 person-years** | **Hazard ratio (95%CI)** | **p-Value** |
| **Oral health problems** | | | | | | |
| Mouth ulcers | 21.19 | 1.296 (1.086-1.548) | **0.0041**** | 39.73 | 1.193 (1.049-1.357) | **0.0071**** |
| Painful gums | 28.11 | 1.449 (1.105-1.899) | **0.0072**** | 42.70 | 1.140 (0.917-1.417) | 0.2372 |
| Bleeding gums | 18.23 | 1.101 (0.930-1.303) | 0.2615 | 33.46 | 0.992 (0.877-1.122) | 0.9032 |
| Loose teeth | 25.86 | 1.239 (0.974-1.576) | 0.0802 | 43.27 | 1.034 (0.860-1.244) | 0.7171 |
| Toothache | 18.03 | 0.970 (0.734-1.281) | 0.8318 | 36.10 | 0.982 (0.807-1.195) | 0.8601 |
| Dentures | 25.69 | 1.308 (1.129-1.515) | **0.0004***** | 47.25 | 1.154 (1.038-1.283) | **0.0080**** |

***Note:*** This analysis used multiple imputation for missing covariates. Data analysed using Cox regression to obtain HR and 95% CI. Model adjusted for age, sex, ethnicity, education level, income level, BMI, smoking status, drinking status, hypertension, diabetes and stroke status. Incidence rate per 100,000 person-years = (Number of cases with a specific oral health problem / Total person-years with that specific oral health problem) × 100000. Bold text indicates statistically significant associations (<0.05).

Abbreviation: HR: Hazard ratio, CI: Confidence interval. BMI: body mass index. *p<0.05, **p<0.01, ***p<0.001.

**Supplementary Table 9. Sensitivity analysis of the number of oral health problems with incident IBD : multiple imputation for missing covariates.**

| **Variable** | **Incident Crohn’s disease** | | | **Incident ulcerative colitis** | | |
| --- | --- | --- | --- | --- | --- | --- |
|  | **Hazard ratio (95%CI)** | **p-Value** | **p-Value for Trend** | **Hazard ratio (95%CI)** | **p-Value** | **p-Value for Trend** |
| **Number of oral problems** |  |  |  |  |  |  |
| No oral health problem | 1 (reference) |  | **1.36×10^-5^***** | 1 (reference) |  | **0.0098**** |
| one oral health problem | 1.159 (1.017-1.320) | **0.0263*** |  | 1.063 (0.971-1.165) | 0.1847 |  |
| two oral health problems | 1.258 (1.016-1.557) | **0.0345*** |  | 1.143 (0.980-1.332) | 0.0869 |  |
| three oral health problems | 1.738 (1.075-2.412) | **0.0018**** |  | 1.364 (1.040-1.790) | **0.0248*** |  |
| More than three oral problems | 2.146 (1.282-3.594) | **0.0037**** |  | 1.117 (0.681-1.832) | 0.6598 |  |

***Note:*** This analysis used multiple imputation for missing covariates. Data analysed using Cox regression to obtain HR and 95% CI. Model adjusted for age, sex, ethnicity, education level, income level, BMI, smoking status, drinking status, hypertension, diabetes and stroke status. Bold text indicates statistically significant associations (<0.05).

Abbreviation: HR: Hazard ratio, CI: Confidence interval. BMI: body mass index. *p<0.05, **p<0.01, ***p<0.001.

**Supplementary Table 10. Mediation analysis of INFLA in the association of oral health problems and Crohn’s disease.**

| **Independent variable** | **Indirect effect** | | **Direct effect** | | **Total effect** | | **Total effect mediated** |
| --- | --- | --- | --- | --- | --- | --- | --- |
|  | **Coefficient** | **95%CI** | **Coefficient** | **95%CI** | **Coefficient** | **95%CI** | **%** |
| oral ulcers | -0.00000626 | (-0.0000152,0.00000149) | 0.000620 | **(0.0000749,0.00119)*** | 0.000613 | **(0.0000723,0.00118)*** | -1.02% |
| painful gums | 0.0000383 | **(0.0000207,0.0000617)***** | 0.00120 | **(0.000184,0.002322)*** | 0.00124 | **(0.000218,0.002378)*** | **3.09%*** |
| dentures | 0.0000295 | **(0.0000199,0.0000399)***** | 0.000514 | **(0.0000876,0.00101)*** | 0.000546 | **(0.000116,0.001031)**** | **5.40%**** |

***Note:*** A total of 377,855 participants were included in the analysis, and models were adjusted for age, sex, ethnicity, education level, income level, BMI, smoking status, drinking status, hypertension, diabetes and stroke status.

Abbreviation: CI: Confidence interval. BMI: body mass index. *p<0.05, **p<0.01, ***p<0.001.

**Supplementary Table 11. Mediation analysis of INFLA in the association of oral health problems and ulcerative colitis.**

| **Independent variable** | **Indirect effect** | | **Direct effect** | | **Total effect** | | **Total effect mediated** |
| --- | --- | --- | --- | --- | --- | --- | --- |
|  | **Coefficient** | **95%CI** | **Coefficient** | **95%CI** | **Coefficient** | **95%CI** | **%** |
| oral ulcers | -0.00000977 | (-0.0000232,0.00000229) | 0.000581 | (-0.000198,0.001441) | 0.000571 | (-0.000207,0.001438) | -1.71% |
| dentures | 0.0000469 | **(0.0000326,0.0000630)***** | 0.000396 | (-0.000206,0.001033) | 0.000442 | (-0.000161,0.001082) | 10.6% |

***Note:*** A total of 377,855 participants were included in the analysis, and models were adjusted for age, sex, ethnicity, education level, income level, BMI, smoking status, drinking status, hypertension, diabetes and stroke status.

Abbreviation: CI: Confidence interval. BMI: body mass index. *p<0.05, **p<0.01, ***p<0.001.

**Supplementary Figure 1. Mediation analysis path diagram.**


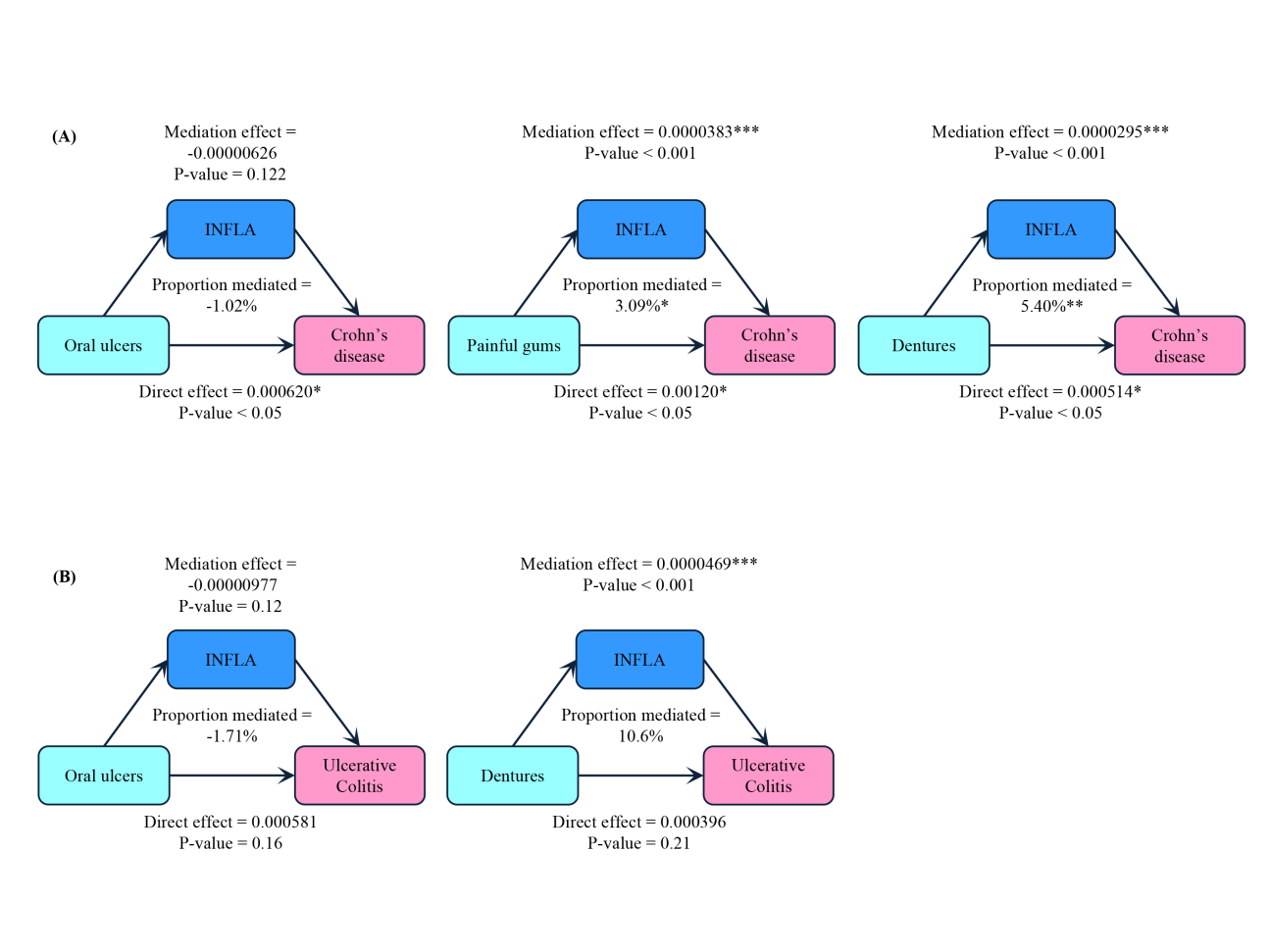


***Note:*** (A) Mediation analysis of INFLA on the association between oral health problems and CD. (B) Mediation analysis of INFLA on the association between oral health problems and UC. The mediation effect indicated whether INFLA was in the pathway between oral health and CD/UC. The proportion mediated = Indirect effect / [Indirect effect + Direct effect]. Adjusted for age, sex, ethnicity, education level, income level, BMI, smoking status, drinking status, hypertension, diabetes and stroke status.

Abbreviations: INFLA: low-grade inflammation index. BMI: body mass index.

**Supplementary Figure 2. Subgroup analysis of the association between mouth ulcers with incident Crohn’s disease.**


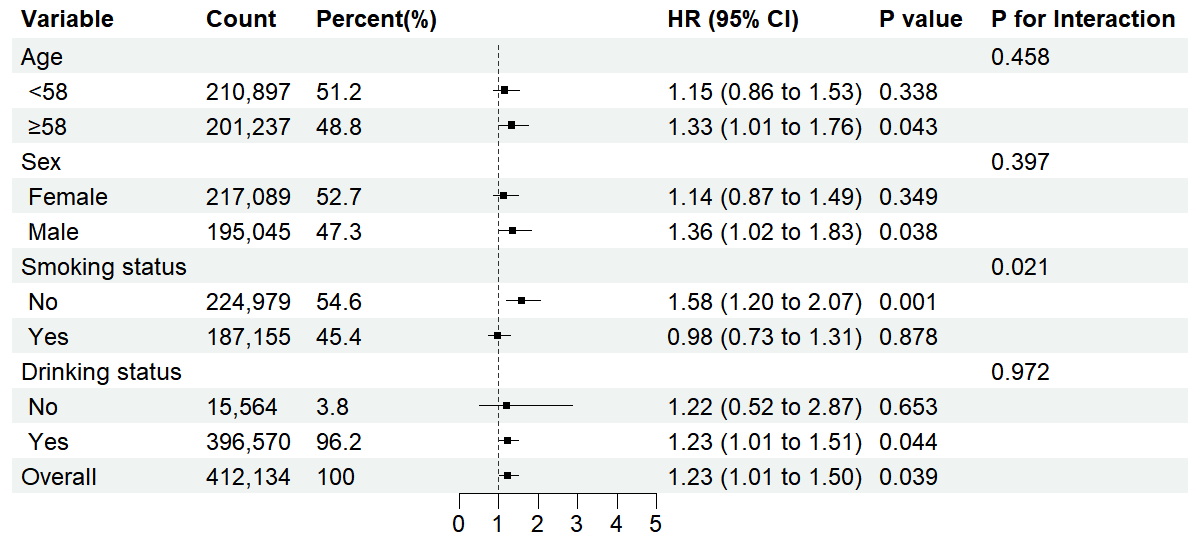


***Note:*** All analyses were performed using Cox regression with models adjusted for baseline age, sex, ethnicity, education level, income level, BMI, smoking status, drinking status, hypertension, diabetes and stroke status.

Abbreviation: HR: Hazard ratio, CI: Confidence interval. BMI: body mass index. Likelihood ratio tests were applied to formally test for interactions.

**Supplementary Figure 3. Subgroup analysis of the association between painful gums with incident Crohn’s disease.**


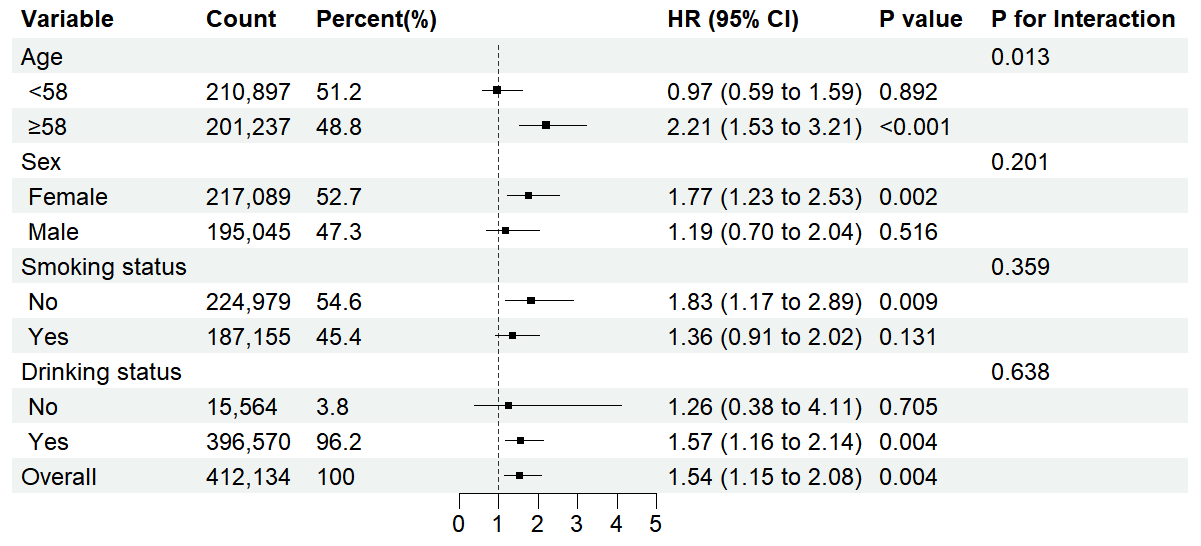


***Note:*** All analyses were performed using Cox regression with models adjusted for baseline age, sex, ethnicity, education level, income level, BMI, smoking status, drinking status, hypertension, diabetes and stroke status.

Abbreviation: HR: Hazard ratio, CI: Confidence interval. BMI: body mass index. Likelihood ratio tests were applied to formally test for interactions.

**Supplementary Figure 4. Subgroup analysis of the association between dentures with incident Crohn’s disease.**


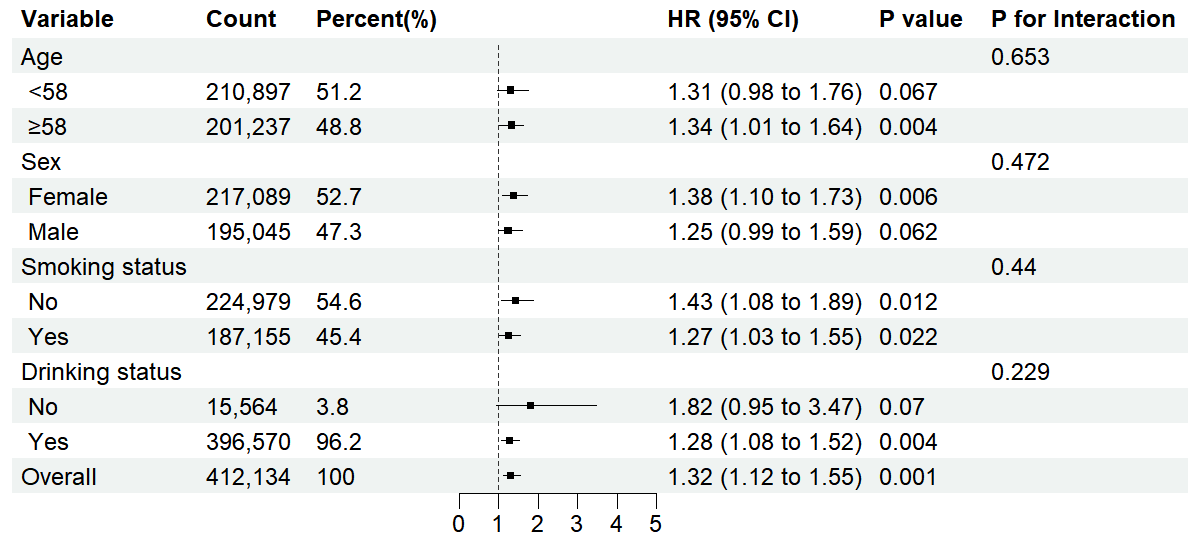


***Note:*** All analyses were performed using Cox regression with models adjusted for baseline age, sex, ethnicity, education level, income level, BMI, smoking status, drinking status, hypertension, diabetes and stroke status.

Abbreviation: HR: Hazard ratio, CI: Confidence interval. BMI: body mass index. Likelihood ratio tests were applied to formally test for interactions.

**Supplementary Figure 5. Subgroup analysis of the association between mouth ulcers with incident ulcerative colitis.**


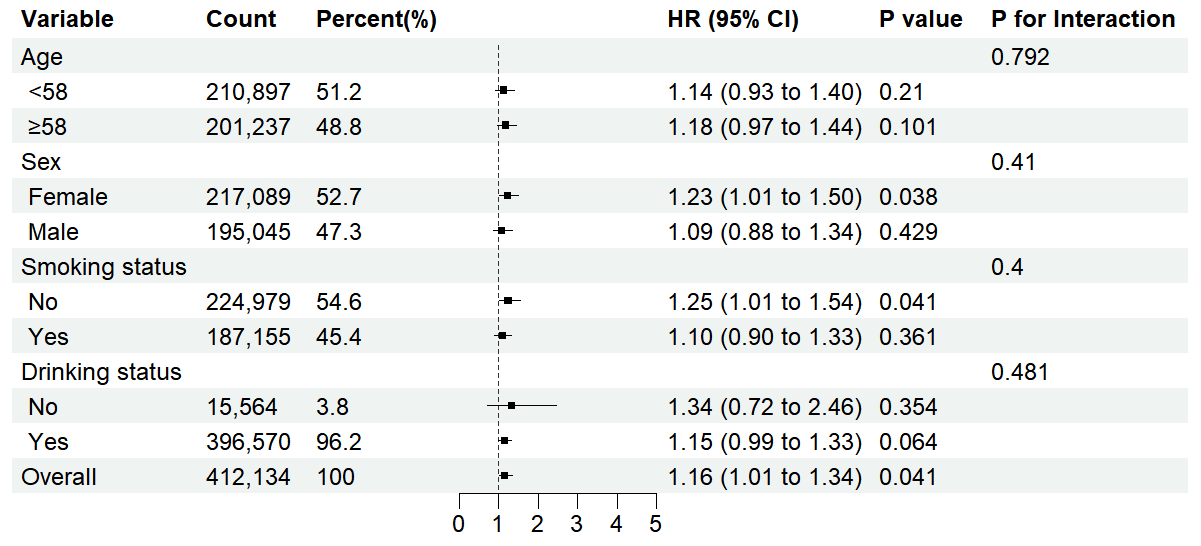


***Note:*** All analyses were performed using Cox regression with models adjusted for baseline age, sex, ethnicity, education level, income level, BMI, smoking status, drinking status, hypertension, diabetes and stroke status.

Abbreviation: HR: Hazard ratio, CI: Confidence interval. BMI: body mass index. Likelihood ratio tests were applied to formally test for interactions.

**Supplementary Figure 6. Subgroup analysis of the association between dentures with incident ulcerative colitis.**


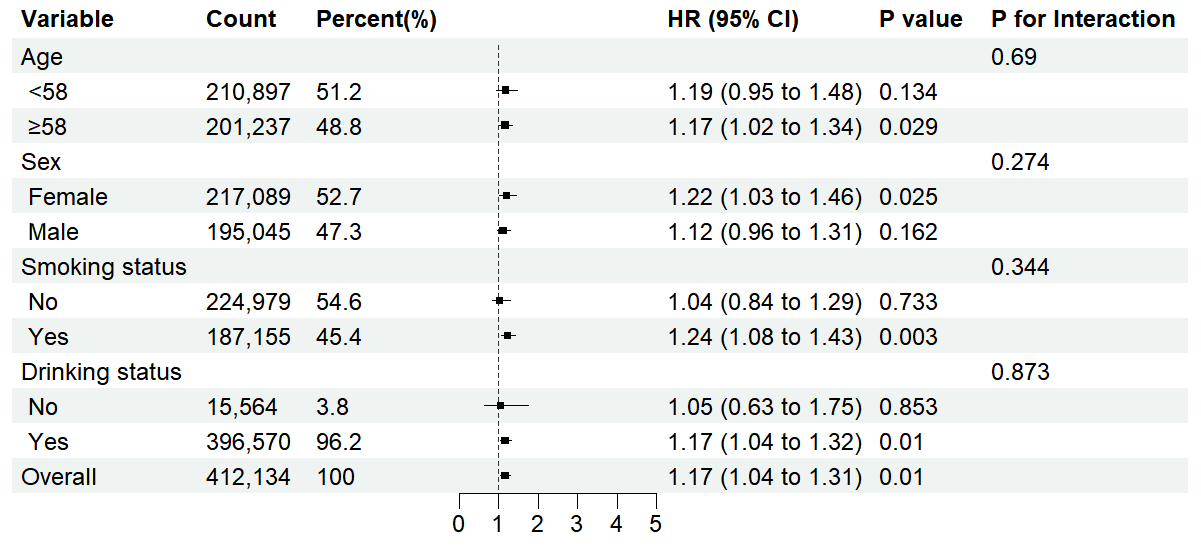


***Note:*** All analyses were performed using Cox regression with models adjusted for baseline age, sex, ethnicity, education level, income level, BMI, smoking status, drinking status, hypertension, diabetes and stroke status.

Abbreviation: HR: Hazard ratio, CI: Confidence interval. BMI: body mass index. Likelihood ratio tests were applied to formally test for interactions.

**Supplementary Methods**

**Covariates**

1. Ethnicity was classified as white, Asian or Asian British, black or black British, and other.
2. Educational level was self-reported by participants and divided into six categories: College or University degree and others
3. The levels of household annual income are categorized into four groups: < £18,000, £18,000-£30,999, £31,000-£51,999, and > £52,000.
4. Body mass index (BMI) is based on the formula (weight (kg) / height (m^2^)) and is divided by WHO into underweight (<18.5 kg/m^2^), normal weight (18.5-24.9 kg/m^2^), overweight (25.0-29.9 kg/m^2^), and obesity (≥30 kg/m^2^).
5. Smoking status was classified into current, former, and never smokers based on self-report.
6. Alcohol consumption was categorized as current, former, and never drinkers based on self-report.
7. Hypertension, diabetes, and stroke were classified as “yes” or “no” based on self-report at baseline. Those with missing covariates above were excluded from this analysis.
8. Age: The age was calculated based on the date of attendance at the assessment center and the date of birth.
9. Sex: We defined sex as a set of biological attributes linked to physical and physiological features, including chromosomes, hormone levels, and anatomy. Typically, we assign a binary sex (male/female) at birth based solely on a newborn's visible external anatomy.
